# Neutrophil Extracellular Traps Formation and Aggregation Orchestrate Induction and Resolution of Sterile Crystal-Mediated Inflammation

**DOI:** 10.3389/fimmu.2018.01559

**Published:** 2018-07-06

**Authors:** Yanhong Li, Xue Cao, Yi Liu, Yi Zhao, Martin Herrmann

**Affiliations:** ^1^Department of Rheumatology and Immunology, West China Hospital, Sichuan University, Chengdu, China; ^2^Department of Internal Medicine 3, Rheumatology and Immunology, Friedrich-Alexander-University Erlangen-Nürnberg (FAU), Erlangen, Germany

**Keywords:** sterile crystal, inflammation, neutrophil, neutrophil extracellular traps, aggregated neutrophil extracellular traps

## Abstract

The formation of neutrophil extracellular traps (NETs) to immobilize pathogens represents a novel antimicrobial strategy of the immune system. The microcrystals related to human diseases are classified into endogenous microcrystals, including monosodium urate (MSU), calcium pyrophosphate dihydrate, calcium carbonate, calcium phosphate, calcium oxalate, cholesterol, and exogenous material like crystals from silica. Although microcrystals possess distinct compositions and shapes, they have a common characteristic: they stimulate neutrophils to release NETs. In low and high densities, neutrophils form NETs and aggregated NETs (aggNETs) that reportedly orchestrate the initiation and resolution of sterile crystal-mediated inflammation, respectively. Here, we summarize the different roles of NETs and aggNETs stimulated by the crystals mentioned above in related inflammatory reactions. The NETosis-derived products may represent a potential therapeutic target in crystal-mediated diseases.

## Introduction

Neutrophils are the most abundant circulated leukocytes in blood and represent the first line of innate immune system to defense against the injury or infection, including bacteria, fungi, and protozoa. In addition to their classical functions like phagocytosis and degranulation, neutrophils are endowed with a novel antimicrobial strategy. They are able to entrap and kill pathogens by extruding their nucleohistone network into the extracellular space, known as neutrophil extracellular traps (NETs) (1). NETs formation can be induced by various kinds of stimuli, such as specific microbes, sterile inflammatory mediators like lipopolysaccharides (LPS), phorbol-12-myristate-13-acetate (PMA), IL-8, ionomycin (2) or cytokines (3), immune complexes, activated platelets (4), particulate matter, and microcrystals (5). Under certain circumstances NETs formation is accompanied by a unique cell death program referred to as NETosis. The latter is distinct from apoptosis and necrosis (1, 6). During NETosis, the chromatin decondenses, the membranes of nucleus and granules disintegrate to generate chromatin blended with nuclear and granular proteins and enzymes. Following the rupture of the cellular membrane, chromatin is decorated with a plethora of internal molecules, including histones, neutrophil elastase (NE), myeloperoxidase (MPO), proteinase-3 (PR3), lactoferrin, cathepsin G, matrix metalloprotease 9, peptidoglycan-recognition proteins, high mobility group protein B1, pentraxin, LL-37, and the bactericidal/permeability-increasing protein (1, 6). These contents are then expelled into the extracellular environment (1, 7–10). However, several aspects of NETs formation still remain elusive. In some forms of NETs formation the production of reactive oxygen species (ROS) by NADPH oxidase or mitochondria emerged to play an integral role; ROS helps to translocate the granular proteins MPO and NE into the nucleus (6, 7). Meanwhile, a number of molecules have been identified contributing to NETs formation, including peptidyl arginine deiminase 4 (PADI4), TGF-β-activated kinase 1, intracellular Ca^2+^, and RAF/MEK/ERK (11). Due to the complicated structure and cytotoxic enzymes, NETs have been implicated in orchestrating the local immune response *via* eliminating pathogens, releasing pro-inflammatory mediators, and damaging tissue directly (12–18). In the pathogenesis of autoimmune inflammatory diseases, such as vasculitis, RA, and SLE they may serve as source of neoantigens that trigger the production of autoantibodies. A number of studies have suggested that NETs are involve in trapping microcrystals when the human body is exposed to crystals from monosodium urate (MSU), calcium pyrophosphate dihydrate (CPP), cholesterol, calcium carbonate (CaCO_3_), calcium phosphate (CaP), calcium oxalate (CaOx), or silica under conditions of sterile inflammation (19). The latent role of NETs in crystal-induced inflammation is either to induce local necroinflammation or to perform a state of alleviating inflammation, just like acute or chronic gout, respectively (20). Herein, we review the current state of knowledge regarding NETs formation and aggregation in sterile inflammation induced by different sizes and shapes of microcrystals.

## Monosodium Urate Crystals

Crystals of MSU monohydrate (NaC_5_H_3_N_4_O_3·_H_2_O) are macroscopically needle-shaped (21, 22). Their deposition in joints and soft tissues can cause an acute, inflammatory joint disease, usually referred to as gouty arthritis (23). In joints, MSU crystals induce the secretion of cytokines and chemokines by phagocytes, including interleukin (IL)-1β, tumor necrosis factor (TNF), IL-6, and IL-8 (24). These inflammatory mediators are crucial to amplify inflammation by the recruitment into the joint synovial fluid of further neutrophils and monocytes (24). MSU crystals are supposed to activate neutrophils to release cytokines and induce infiltration of further neutrophils to form NETs (25, 26) leading to acute, extremely painful, and tissue-damaging inflammation in joints.

The NET release from neutrophils induced by MSU crystals is a complex yet highly coordinated sequence of events. To facilitate this, a lot of signaling pathways have been evolved. It was reported that NADPH oxidase-mediated ROS production is required for NETs formation evoked by MSU crystals (19, 27). Neutrophils from patients with chronic granulomatous disease (CGD) or NADPH oxidase-deficient mice displayed weak NETs formation in response to MSU (19, 27). *In vitro*, neutrophils treated with various anti-oxidants, lack ROS production and NETs formation in response to MSU crystals (19, 27). Furthermore, NETs formation induced by soluble uric acid is mediated by NF-κB activation is independent of ROS production (28). Unexpectedly, there is report that MSU crystals induce NETosis in an NADPH oxidase-independent fashion distinct from PMA-induced NETosis (29). Also, autophagy, necroptosis, RIPK1-RIPK3-MLKL signaling, and endosomal acidification have emerged as key regulators of MSU crystal-induced NETs formation (20, 30). The stress-related protein REDD1 which expressed in neutrophils is regulated in development and DNA damage responses. At the attack phase of familial Mediterranean fever (FMF), upregulated REDD1 promote autophagy and augment NETs formation (31). Consistently, during remission phases of FMF, the transcription of REDD1 is impaired and resistant to autophagy-mediated NETs release (31). Specifically, blocking phosphatidylinositol 3-kinase signaling or phagolysosome fusion prevents MSU crystal-induced NETs formation (26, 30). Result from RIPK3 knockout murine demonstrated that neutrophils deficient in RIPK3 are unable to release NETs in response to MSU crystal *in vitro* or *in vivo* (26). By contrast, other studies showed that PMA stimulates NETs release independent of RIPK 3 and MLKL signaling (32). Studies have described substantial effects of the purinergic receptors, P2Y and P2Y6 on MSU crystal-induced NETs formation (33). MRS2578, an inhibitor of P2Y6, reportedly restrained neutrophil migration and production of ROS as shown by live cell imaging. This suggests that purinergic receptors are involved in NETs formation (33). Interestingly, SK&F96365 inhibited MSU crystal-induced NETs formation by affecting a store-operated calcium entry channel (33). Neutrophils and phagocytes internalize small urate micro-aggregates (UMA; <1μm in size) in the circulation and thus suppress MSU crystals formation (34). Gradually, mass of urate micro-aggregates exceeds the phagocytic ability of neutrophils leading to MSU crystals formation. Consequently, neutrophils are frustrated in phagocytosis and generate NETs (34, 35).

The NETs formation in response to MSU crystals is a complex process modulated by a plethora of factors, including inflammatory cytokines. In a MSU crystal-induced arthritis model has been shown that IL-1 inhibition is effective to control MSU-mediated inflammation (36). Neutrophil exhibit an enhanced NETs release in response to synovial fluid from gout patients, partially hindered by the IL-1β antagonist anakinra (30) and IL-1β accelerates NETs formation triggered by MSU crystals (37).

Interestingly, IL-1β alone does not stimulate NETs release (37) and positive feedback loops are crucial for NETs formation. In joints NETs release dangerous neutrophilic cargoes like histones and granule proteins like myeloperoxidase (MPO) and NE (1) further amplifying MSU crystal-induced inflammation. In addition, NE cleaves pro-IL-1β into its bioactive IL-1β and IL-1β is a crucial cytokine of the inflammatory response in gout. It recruits neutrophils to joints and enhances NETs formation triggered by MSU crystals (37–39). Apart from that, DAMPs released from NETs, such as DNA-activating Toll-like receptors or NLRP3 inflammasomes can augment inflammatory responses (40). MSU crystals induce NETosis through neutrophils in patients with gout. NETosis is further enhanced by impaired NETs degradation result from low DNase-1 activity in synovial fluids, in conjunction with enriched actin that is resistant to DNase degradation (29, 40). The aberrant accumulation of aggregated NETs (aggNETs) is associated with NETs clearance deficiency and formation of extended NETs-crystal aggregates (40). In high neutrophil densities, NETs will agglomerate and form aggNETs (19).

**Figure 1:**
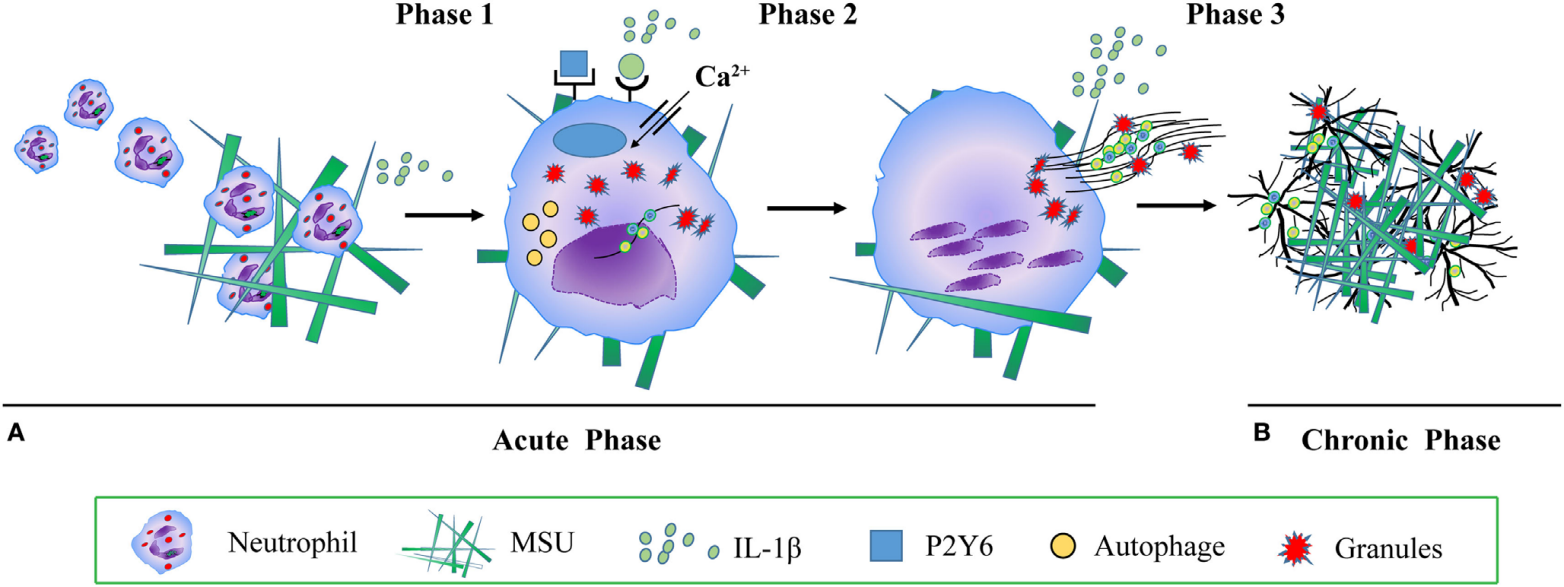
The molecular mechanism of monosodium urate (MSU) crystals induced neutrophil extracellular traps (NETs) and aggregated NETs (aggNETs) formation. **(A)** Acute gouty attack: phase 1 MSU crystals induce mononuclear phagocytes to secrete cytokines and chemokines, including interleukin (IL)-1β, tumor necrosis factor, IL-6, and IL-8. IL-1β recruits further neutrophils into the synovial joint; phase 2 neutrophils take up MSU crystals, a process activating reactive oxygen species, autophagy, RIPK1-RIPK3-MLKL signaling, P2Y6 receptor, endosomal acidification, calcium entry channel, IL-1β, and inducing NETs formation. NETs release dangerous neutrophils’ cargo, such as histones, MPO, NE, cytokine, DAMPs, chemokines, leading to acute, painful, and tissue-damaging inflammation; **(B)** chronic tophaceous gout: phase 3 under conditions of high neutrophil densities, aggNETs are formed, degrade pro-inflammatory chemokines and cytokines, and suppress crystal-induced inflammation. Abbreviations: MPO, myeloperoxidase; NE, neutrophil elastase; DAMPs, danger associated molecular patterns.

It has been shown that aggNETs degrade pro-inflammatory chemokines and cytokines and suppress crystal-induced inflammation (19) *in vitro* and *in vivo*. Animal studies suggest an important anti-inflammatory role of aggNETs in the regulation of cytokines like TNF-α, IL-1β and IL-6, and chemokines, such as chemokine ligand CCL2 and monocyte chemoattractant protein-1 (19). Collectively, these findings suggest that aggNETs promote the resolution of acute gouty arthritis (19). Despite its important role in the resolution of inflammation, little is known about the regulation of aggNETs formation. It has been reported that MSU crystals induced aggNETs formation depends on the production of ROS (19). *In vitro*, neutrophils of patients with CGD co-cultured with MSU crystals show reduced formation of aggNETs (19). *In vivo*, NADPH oxidase-deficient mice reduced the formation of aggNETs, when stimulated with MSU, both in an air pouch model, and in MSU crystal-induced paw inflammation (19). Accordingly, the authors also reported that ATP, lactoferrin (19), IL-1β (37) and the P2Y6 receptor antagonist MRS2578 (33) enhance and inhibit MSU crystal-induced aggNETs formation, respectively (Figure 1).

## Calcium Pyrophosphate Dihydrate Crystals

Calcium pyrophosphate dihydrate (CCP) crystals display a rhomboid or elongated shape, and are generally shorter than 10 μm in length (41). They tend to deposit in joints and causes pseudogout, characterized by periodic acute joint flares (42). CPP crystals directly interact with and activate macrophages to release IL-1β, which attract more neutrophils to the inflamed joints (43–46). Neutrophils phagocytosis of CPP crystals resulted in the release of NETs composed of extracellular DNA colocalizing with citrullinated histone H4 and myeloperoxidase (MPO) (47). CPP crystals elicited NETs faster and more efficiently than PMA (47). Going along with nuclear DNA morphological changes, neutrophils execute an NADPH oxidase-independent process of NETs release (47). The activity of the ERK/MEK signaling pathway, heat shock protein 90, and PI3K are essential for NETs formation triggered by CPP crystals (47). An intact cytoskeleton is required for CPP crystal-promoted NETs extrusion (47) and the release of IL-1β, IL-8, TNF-α, and GM-CSF from macrophages and neutrophils are important driving forces that promote NETs formation (47). In turn, NETs release ROS, MPO, DNA, IL-1β, and IL-8, and thus further activate inflammation, which can damage the joints (47).

## Cholesterol Crystals

Cholesterol is a lipid of endogenous or environmental origin. When the temperature in plasma is under 37°C, circulating cholesterol will form monohydrate cholesterol crystals which foster the formation of atherosclerotic plaques (48). In addition to the crystallization of cholesterol in the circulation the release of IL-1β may activate the IL-1receptors (IL-1R) on the surface of neutrophils to further enhance the migration of neutrophils into the site of atherosclerotic lesions (49–53). The first observation that cholesterol crystals trigger NETs formation reported that translocation of ROS, NE, and PR3 into the nucleus are required for cholesterol crystals to induce NETs release (54). Interestingly, inhibiting peptidyl arginine deiminase enzymes did not affect NETs formation triggered by cholesterol crystals (54), whereas NE and PR3-deficient mice do not form NETs in an atherosclerosis model and exhibited a reduced plaque size relative to controls (54). NETs augment the transcription of pro-inflammatory cytokines. In co-cultures with cholesterol crystals, NETs can enhance the cytokines released from macrophage *via* the IL-1/IL-17 loop (54). NETs and cholesterol crystals can also activate Th17 cells to sustain a chronic sterile inflammation (54). Overall, NETs release modulated the inflammation in atherosclerotic plaques.

## Calcium Carbonate Crystals

Calcium carbonate—a white and tasteless microcrystalline powder—is generally less than 1 μm in diameter (54) and possess different shapes encompassing layered, rhombohedral, irregular, needlelike, spherulitic, or cubelike shape according to various polymorphic crystalline phases (55). As the component of pancreatic juice, calcium carbonate crystals can induce NETs and aggregate NETs formation (56, 57). Ductal occlusion is a main cause in the pathogenesis of pancreatitis; however, the mechanism of action remains elusive. Samples of histological tissue sections and pancreatic juice from patients with pancreatitis show aggNETs, as a specific occluding agent that agglomerate in the pancreatic ducts (58). Further studies confirmed that bicarbonate ions and calcium carbonate crystals can both induce aggNETs formation in the ductal tree during pancreatitis *via* a PADI4-dependent signaling pathway (58, 59).

## Silica Crystals

Silica crystals, also termed crystalline silica or silicon dioxide (SiO_2_), are usually harmless and non-toxic crystals; however, inhalation of silica can cause pulmonary inflammation (58). Prolonged exposure to silica crystals, especially for coal-miners and smokers, confers a great risk to develop pneumoconiosis silicosis, a chronic, progressive, irreversible, and incurable disease characterized by pulmonary fibrosis. Therefore, the understanding of the mechanisms of silica-induced pulmonary fibrosis is indispensably. After the interaction with silica, alveolar macrophages and endothelial cells (60) ingest silica, activate the NALP3 inflammasome (61), produce ROS and lead to phagolysosomal damage (60). Then massive chemokines and pro-inflammatory cytokines like IL-1β and TNF-α are generated, neutrophils are recruited to lung tissue and assist in the clearance of the silica crystals (60). In addition to phagocytic clearance, neutrophils undergo NETosis (60, 62–64) is an important factor in the establishment of the lung disease (65).

At onset of the diseases, NETs are scarce and release numerous pro-inflammatory cytokines and cytotoxic contents which promote the acute inflammation (20). Along with the disease progression, aggNETs are formed and showed an anti-inflammatory effect on the local microenvironment by eliminating pro-inflammatory mediators, trapping silica coated with proteases and sequester them in silicotic nodules, which resemble gouty tophi (20). The persistence of silicotic nodules is prone to sustain inflammation and may cause silicosis or even lung cancer (60).

## Further Crystalline Materials

There is evidence that CaP and CaOx may trigger RIPK1-RIPK3-MLKL-dependent neutrophil necroptosis and promote NETs or aggNETs formation *in vitro* and *in vivo* (20). When exposed to CaP, IL-1β derived from macrophages activates NETs formation which in turn induces the secretion of TNF-α by macrophages (66). Further studies on NETs in CaP or CaOx related to diseases like nephrolithiasis are needed.

## Conclusion

Sterile crystal-mediated diseases, such as gout, pseudogout, atherosclerosis, and silicosis are highly prevalent worldwide. As summarized here, NETs are often involved in the progression of these kinds of diseases where they play detrimental and also beneficial roles. The activation of the NALP3 inflammasome, production of ROS, and the pro-inflammatory cytokines IL-1β, TNF-α are critical for NETosis in crystal-induced inflammation. NETs and aggNETs govern acute and chronic phase of sterile inflammation, respectively. Finally, targeting NETosis and NETs-derived products may provide new therapeutic approaches for crystal-mediated diseases.

## Author Contributions

YL and XC contributed equally to this article. YL, XC, and YZ wrote the first draft of this article. YL, XC, and YZ designed the figures. YL and MH critically revised the manuscript for important intellectual content. All authors approved the final version.

## Conflict of Interest Statement

The authors declare that the research was conducted in the absence of any commercial or financial relationships that could be construed as a potential conflict of interest.

## References

[1] Brinkmann V Reichard U Goosmann C Fauler B Uhlemann Y Weiss DS Neutrophil extracellular traps kill bacteria. Science (2004) 303(5663):1532–5. 10.1126/science.1092385 15001782

[2] Parker H Dragunow M Hampton MB Kettle AJ Winterbourn CC. Requirements for NADPH oxidase and myeloperoxidase in neutrophil extracellular trap formation differ depending on the stimulus. J Leukoc Biol (2012) 92(4):841–9. 10.1189/jlb.1211601 22802447

[3] Keshari RS Jyoti A Dubey M Kothari N Kohli M Bogra J Cytokines induced neutrophil extracellular traps formation: implication for the inflammatory disease condition. PLoS One (2012) 7(10):e48111. 10.1371/journal.pone.0048111 23110185 PMC3482178

[4] Carestia A Kaufman T Schattner M. Platelets: new bricks in the building of neutrophil extracellular traps. Front Immunol (2016) 7:271. 10.3389/fimmu.2016.00271 27458459 PMC4933697

[5] Sørensen OE Borregaard N. Neutrophil extracellular traps – the dark side of neutrophils. J Clin Invest (2016) 126(5):1612. 10.1172/JCI84538 27135878 PMC4855925

[6] Steinberg BE Grinstein S. Unconventional roles of the NADPH oxidase: signaling, ion homeostasis, and cell death. Sci STKE (2007) 2007(379):e11. 10.1126/stke.3792007pe11 17392241

[7] Fuchs TA Abed U Goosmann C Hurwitz R Schulze I Wahn V Novel cell death program leads to neutrophil extracellular traps. J Cell Biol (2007) 176(2):231. 10.1083/jcb.200606027 17210947 PMC2063942

[8] Kolaczkowska E Kubes P. Neutrophil recruitment and function in health and inflammation. Nat Rev Immunol (2013) 13(3):159. 10.1038/nri3399 23435331

[9] Jaillon S Peri G Delneste Y Frémaux I Doni A Moalli F The humoral pattern recognition receptor PTX3 is stored in neutrophil granules and localizes in extracellular traps. J Exp Med (2007) 204(4):793–804. 10.1084/jem.20061301 17389238 PMC2118544

[10] Kessenbrock K Krumbholz M Schönermarck U Back W Gross WL Werb Z Netting neutrophils in autoimmune small-vessel vasculitis. Nat Med (2009) 15(6):623. 10.1038/nm.1959 19448636 PMC2760083

[11] Metzler KD Goosmann C Lubojemska A Zychlinsky A Papayannopoulos V. A myeloperoxidase-containing complex regulates neutrophil elastase release and actin dynamics during NETosis. Cell Rep (2014) 8(3):883–96. 10.1016/j.celrep.2014.06.044 25066128 PMC4471680

[12] Wang Y Li M Stadler S Correll S Li P Wang D Histone hypercitrullination mediates chromatin decondensation and neutrophil extracellular trap formation. J Cell Biol (2009) 184(2):205. 10.1083/jcb.200806072 19153223 PMC2654299

[13] Leshner M Wang S Lewis C Zheng H Chen XA Santy L PAD4 mediated histone hypercitrullination induces heterochromatin decondensation and chromatin unfolding to form neutrophil extracellular trap-like structures. Front Immunol (2012) 3(2–3):307. 10.3389/fimmu.2012.00307 23060885 PMC3463874

[14] Hakkim A Fuchs TA Martinez NE Hess S Prinz H Zychlinsky A Activation of the Raf-MEK-ERK pathway is required for neutrophil extracellular trap formation. Nat Chem Biol (2011) 7(2):75. 10.1038/nchembio.496 21170021

[15] Keshari RS Verma A Barthwal MK Dikshit M Reactive oxygen species-induced activation of ERK and p38 MAPK mediates PMA-induced NETs release from human neutrophils. J Cell Biochem (2013) 114(3):532–40. 10.1002/jcb.24391 22961925

[16] Alemán OR Mora N Cortes-Vieyra R Uribe-Querol E Rosales C Transforming growth factor-β-activated kinase 1 is required for human FcγRIIIb-induced neutrophil extracellular trap formation. Front Immunol (2016) 7:277 10.3389/fimmu.2016.00277 27486461 PMC4947870

[17] Demaurex N Monod A Lew DP Krause KH. Characterization of receptor-mediated and store-regulated Ca2+ influx in human neutrophils. Biochem J (1994) 297(Pt 3):595. 10.1042/bj2970595 8110199 PMC1137875

[18] Behnen M Leschczyk C Möller S Batel T Klinger M Solbach W Immobilized immune complexes induce neutrophil extracellular trap release by human neutrophil granulocytes via FcγRIIIB and Mac-1. J Immunol (2014) 193(4):1954–65. 10.4049/jimmunol.1400478 25024378

[19] Schauer C Janko C Munoz LE Zhao Y Kienhöfer D Frey B Aggregated neutrophil extracellular traps limit inflammation by degrading cytokines and chemokines. Nat Med (2014) 20(5):511. 10.1038/nm.3547 24784231

[20] Desai J Foresto-Neto O Honarpisheh M Steiger S Nakazawa D Popper B Particles of different sizes and shapes induce neutrophil necroptosis followed by the release of neutrophil extracellular trap-like chromatin. Sci Rep (2017) 7(1):15003. 10.1038/s41598-017-15106-0 29101355 PMC5670218

[21] Mandel NS Mandel GS Monosodium urate monohydrate, the gout culprit. J Am Chem Soc (1976) 98(8):2319–23. 10.1021/ja00424a054 1254868

[22] Perrin CM Dobish MA Keuren EV Swift JA Monosodium urate monohydrate crystallization. CrystEngComm (2011) 13(4):1111–7. 10.1039/c0ce00737d

[23] Kuo CF Grainge MJ Zhang W Doherty M. Global epidemiology of gout: prevalence, incidence and risk factors. Nat Rev Rheumatol (2015) 11(11):649. 10.1038/nrrheum.2015.91 26150127

[24] Martin WJ Harper JL. Innate inflammation and resolution in acute gout. Immunol Cell Biol (2010) 88(1):15–9. 10.1038/icb.2009.89 19935764

[25] Hahn J Knopf J Maueröder C Kienhöfer D Leppkes M Herrmann M. Neutrophils and neutrophil extracellular traps orchestrate initiation and resolution of inflammation. Clin Exp Rheumatol (2016) 34(4 Suppl 98):6. 27586795

[26] Desai J Kumar SV Mulay SR Konrad L Romoli S Schauer C PMA and crystal-induced neutrophil extracellular trap formation involves RIPK1-RIPK3-MLKL signaling. Eur J Immunol (2016) 46(1):223–9. 10.1002/eji.201545605 26531064

[27] Schorn C Janko C Krenn V Zhao Y Munoz LE Schett G Bonding the foe – NETting neutrophils immobilize the pro-inflammatory monosodium urate crystals. Front Immunol (2012) 3:376 10.3389/fimmu.2012.00376 23233855 PMC3517988

[28] Arai Y Nishinaka Y Arai T Morita M Mizugishi K Adachi S Uric acid induces NADPH oxidase-independent neutrophil extracellular trap formation. Biochem Biophys Res Commun (2014) 443(2):556. 10.1016/j.bbrc.2013.12.007 24326071

[29] Chatfield SM Grebe K Whitehead LW Rogers KL Nebl T Murphy JM Monosodium urate crystals generate nuclease-resistant neutrophil extracellular traps via a distinct molecular pathway. J Immunol (2018) 200(5):1802–1816. 10.4049/jimmunol.1701382 29367211

[30] Mitroulis I Kambas K Chrysanthopoulou A Skendros P Apostolidou E Kourtzelis I Neutrophil extracellular trap formation is associated with IL-1β and autophagy-related signaling in gout. PLoS One (2011) 6(12):e29318. 10.1371/journal.pone.0029318 22195044 PMC3241704

[31] Skendros P Chrysanthopoulou A Rousset F Kambas K Arampatzioglou A Mitsios A REDD1 links stress with IL-1β-mediated familial Mediterranean fever attack through autophagy-driven neutrophil extracellular traps. J Allergy Clin Immunol (2017) 140(5):1378–87. 10.1016/j.jaci.2017.02.021 28342915

[32] Amini P Stojkov D Wang X Wicki S Kaufmann T Wong WW NET formation can occur independently of RIPK3 and MLKL signaling. Eur J Immunol (2016) 46(1):178–84. 10.1002/eji.201545615 26549703 PMC4738457

[33] Sil P Hayes CP Reaves BJ Breen P Quinn S Sokolove J P2Y6 receptor antagonist MRS2578 inhibits neutrophil activation and aggregated neutrophil extracellular trap formation induced by gout-associated monosodium urate crystals. J Immunol (2017) 198(1):428–42. 10.4049/jimmunol.1600766 27903742

[34] Pieterse E Jeremic I Czegley C Weidner D Biermann MH Veissi S Blood-borne phagocytes internalize urate microaggregates and prevent intravascular NETosis by urate crystals. Sci Rep (2016) 6:38229. 10.1038/srep38229 27917897 PMC5137018

[35] Manfredi AA Ramirez GA Rovere-Querini P Maugeri N. The neutrophil’s choice: phagocytose vs make neutrophil extracellular traps. Front Immunol (2018) 9:288. 10.3389/fimmu.2018.00288 29515586 PMC5826238

[36] Torres R Macdonald L Croll SD Reinhardt J Dore A Stevens S Hyperalgesia, synovitis and multiple biomarkers of inflammation are suppressed by interleukin 1 inhibition in a novel animal model of gouty arthritis. Ann Rheum Dis (2009) 68(10):1602–8. 10.1136/ard.2009.109355 19528034

[37] Sil P Wicklum H Surell C Rada B Macrophage-derived IL-1β enhances monosodium urate crystal-triggered NET formation. Inflamm Res (2017) 66(3):227–37. 10.1007/s00011-016-1008-0 27853847 PMC5296223

[38] Schett G Dayer JM Manger B. Interleukin-1 function and role in rheumatic disease. Nat Rev Rheumatol (2016) 12(1):14–24. 10.1038/nrrheum.2016.166 26656658

[39] Amaral FA Costa VV Tavares LD Sachs D Coelho FM Fagundes CT NLRP3 inflammasome-mediated neutrophil recruitment and hypernociception depend on leukotriene B(4) in a murine model of gout. Arthritis Rheum (2012) 64(2):474. 10.1002/art.33355 21952942

[40] Franklin BS Mangan MS Latz E Crystal formation in inflammation. Annu Rev Immunol (2016) 34(1):173 10.1146/annurev-immunol-041015-055539 26772211

[41] Burt HM Jackson JK Rowell J. Calcium pyrophosphate and monosodium urate crystal interactions with neutrophils: effect of crystal size and lipoprotein binding to crystals. J Rheumatol (1989) 16(6):809–17. 2550631

[42] Liubryan R Lioté F Monosodium urate and calcium pyrophosphate dihydrate (CPPD) crystals, inflammation, and cellular signaling. Joint Bone Spine (2005) 72(4):295–302. 10.1016/j.jbspin.2004.12.010 15990350

[43] Martinon F Pétrilli V Mayor A Tardivel A Tschopp J. Gout-associated uric acid crystals activate the NALP3 inflammasome. Nature (2006) 440(7081):237–41. 10.1038/nature04516 16407889

[44] Pétrilli V Martinon F. The inflammasome, autoinflammatory diseases, and gout. Joint Bone Spine (2007) 74(6):571–6. 10.1016/j.jbspin.2007.04.004 17714972

[45] Martinon F Glimcher LH. Gout: new insights into an old disease. J Clin Invest (2006) 116(8):2073–5. 10.1172/JCI29404 16886051 PMC1523387

[46] Oliveira SHP Canetti C Ribeiro RA Cunha FQ Neutrophil migration induced by IL-1β depends upon LTB4 released by macrophages and upon TNF-α and IL-1β released by mast cells. Inflammation (2008) 31(1):36–46. 10.1007/s10753-007-9047-x 17874178

[47] Pang L Hayes CP Buac K Yoo DG Rada B. Pseudogout-associated inflammatory calcium pyrophosphate dihydrate microcrystals induce formation of neutrophil extracellular traps. J Immunol (2013) 190(12):6488. 10.4049/jimmunol.1203215 23677474

[48] Small DM. George lyman duff memorial lecture. Progression and regression of atherosclerotic lesions. Insights from lipid physical biochemistry. Arteriosclerosis (1988) 8(2):103–29. 10.1161/01.ATV.8.2.103 3348756

[49] Lessner SM Prado HL Waller EK Galis ZS. Atherosclerotic lesions grow through recruitment and proliferation of circulating monocytes in a murine model. Am J Pathol (2002) 160(6):2145–55. 10.1016/S0002-9440(10)61163-7 12057918 PMC1850830

[50] Swirski FK Pittet MJ Kircher MF Aikawa E Jaffer FA Libby P Monocyte accumulation in mouse atherogenesis is progressive and proportional to extent of disease. Proc Natl Acad Sci U S A (2006) 103(27):10340–5. 10.1073/pnas.0604260103 16801531 PMC1502459

[51] Landsman L Bar-On L Zernecke A Kim KW Krauthgamer R Shagdarsuren E CX3CR1 is required for monocyte homeostasis and atherogenesis by promoting cell survival. Blood (2009) 113(4):963. 10.1182/blood-2008-07-170787 18971423

[52] Duewell P Kono H Rayner KJ Sirois CM Vladimer G Bauernfeind FG NLRP3 inflammasomes are required for atherogenesis and activated by cholesterol crystals. Nature (2010) 464(7293):1357–61. 10.1038/nature08938 20428172 PMC2946640

[53] Grebe A Latz E. Cholesterol crystals and inflammation. Curr Rheumatol Rep (2013) 15(3):313. 10.1007/s11926-012-0313-z 23412688 PMC3623938

[54] Warnatsch A Ioannou M Wang Q Papayannopoulos V. Inflammation. Neutrophil extracellular traps license macrophages for cytokine production in atherosclerosis. Science (2015) 349(6245):316–20. 10.1126/science.aaa8064 26185250 PMC4854322

[55] Cizer Ö Rodriguez-Navarro C Ruiz-Agudo E Elsen J Gemert DV Balen KV Phase and morphology evolution of calcium carbonate precipitated by carbonation of hydrated lime. J Sci Mater (2012) 47(16):6151–65. 10.1007/s10853-012-6535-7

[56] Tong H Ma W Wang L Wan P Hu J Cao L. Control over the crystal phase, shape, size and aggregation of calcium carbonate via a L-aspartic acid inducing process. Biomaterials (2004) 25(17):3923. 10.1016/j.biomaterials.2003.10.038 15020169

[57] Beck R Andreassen JP Influence of crystallization conditions on crystal morphology and size of CaCO3 and their effect on pressure filtration. Aiche J (2011) 58(1):107–21. 10.1002/aic.12566

[58] Leppkes M Maueröder C Hirth S Nowecki S Günther C Billmeier U Externalized decondensed neutrophil chromatin occludes pancreatic ducts and drives pancreatitis. Nat Commun (2016) 7:10973. 10.1038/ncomms10973 26964500 PMC4793047

[59] Bilyy R Fedorov V Vovk V Leppkes M Dumych T Chopyak V Neutrophil extracellular traps form a Barrier between necrotic and viable areas in acute abdominal inflammation. Front Immunol (2016) 7(5):424 10.3389/fimmu.2016.00424 27777576 PMC5056318

[60] Hornung V Bauernfeind F Halle A Samstad EO Kono H Rock KL Silica crystals and aluminum salts mediate NALP-3 inflammasome activation via phagosomal destabilization. Nat Immunol (2008) 9(8):847 10.1038/ni.1631 18604214 PMC2834784

[61] Hari A Zhang Y Tu Z Detampel P Stenner M Ganguly A Activation of NLRP3 inflammasome by crystalline structures via cell surface contact. Sci Rep (2014) 4(7281):7281. 10.1038/srep07281 25445147 PMC4250918

[62] Lo Re S Dumoutier L Couillin I Van Vyve C Yakoub Y Uwambayinema F IL-17A-producing gammadelta T and Th17 lymphocytes mediate lung inflammation but not fibrosis in experimental silicosis. J Immunol (2010) 184(11):6367–77. 10.4049/jimmunol.0900459 20421647

[63] Borges VM Lopes MF Falcão H Leite-Júnior JH Rocco PR Davidson WF Apoptosis underlies immunopathogenic mechanisms in acute silicosis. Am J Respir Cell Mol Biol (2002) 27(1):78. 10.1165/ajrcmb.27.1.4717 12091249

[64] Zhai R Ge X Li H Tang Z Liao R Kleinjans J. Differences in cellular and inflammatory cytokine profiles in the bronchoalveolar lavage fluid in bagassosis and silicosis. Am J Ind Med (2004) 46(4):338–44. 10.1002/ajim.20051 15376226

[65] Brinkmann V Goosmann C Kuhn LI Zychlinsky A Automatic quantification of in vitro NET formation. Front Immunol (2012) 3:413 10.3389/fimmu.2012.00413 23316198 PMC3540390

[66] Peng HH Liu YJ Ojcius DM Lee CM Chen RH Huang PR Mineral particles stimulate innate immunity through neutrophil extracellular traps containing HMGB1. Sci Rep (2017) 7(1):16628. 10.1038/s41598-017-16778-4 29192209 PMC5709501

